# Egg White Protein–Proanthocyanin Complexes Stabilized Emulsions: Investigation of Physical Stability, Digestion Kinetics, and Free Fatty Acid Release Dynamics

**DOI:** 10.3390/molecules29030743

**Published:** 2024-02-05

**Authors:** Ting Zhang, Shanglin Li, Meng Yang, Yajuan Li, Xuanting Liu, Xiaomin Shang, Jingbo Liu, Zhiyang Du, Ting Yu

**Affiliations:** 1 Jilin Provincial Key Laboratory of Nutrition and Functional Food, College of Food Science and Engineering, Jilin University, Changchun 130062, China; tingzhang@jlu.edu.cn (T.Z.); lsl19980207@163.com (S.L.); 13756041377@163.com (M.Y.); yiyi_muzi@163.com (Y.L.); lxt920523@163.com (X.L.); xmshang@jlu.edu.cn (X.S.); ljb168@sohu.com (J.L.); dzy2635@163.com (Z.D.); 2 Department of Nutrition, The Second Hospital of Jilin University, Changchun 130041, China

**Keywords:** biomacromolecules, proanthocyanins, structure, emulsion, digestion kinetics

## Abstract

Egg white proteins pose notable limitations in emulsion applications due to their inadequate wettability and interfacial instability. Polyphenol-driven alterations in proteins serve as an effective strategy for optimizing their properties. Herein, covalent and non-covalent complexes of egg white proteins-proanthocyanins were synthesized. The analysis of structural alterations, amino acid side chains and wettability was performed. The superior wettability (80.00° ± 2.23°) and rigid structure (2.95 GPa) of covalent complexes established favorable conditions for their utilization in emulsions. Furthermore, stability evaluation, digestion kinetics, free fatty acid (FFA) release kinetics, and correlation analysis were explored to unravel the impact of covalent and non-covalent modification on emulsion stability, dynamic digestion process, and interlinkages. Emulsion stabilized by covalent complex exhibited exceptional stabilization properties, and FFA release kinetics followed both first-order and Korsmeyer–Peppas models. This study offers valuable insights into the application of complexes of proteins-polyphenols in emulsion systems and introduces an innovative approach for analyzing the dynamics of the emulsion digestion process.

## 1. Introduction

Proteins’ exceptional conformational regulatory properties are instrumental in the emulsification process [1]. Egg white proteins feature outstanding processing adaptability (foaming and gelling properties), which are extensively harnessed in the food industry for crafting puddings, cakes and sausages [2,3]. Egg white proteins, in their unique globular conformation, jointly have both hydrophilic and hydrophobic sites on their surfaces, which potentially facilitate their rapid adsorption at the oil–water interface and inhibit droplet aggregation through the formation of an interfacial film, accompanied by the initiation of electrostatic interactions and steric repulsion [4]. However, the majority of the hydrophobic groups within egg white proteins remain internal to the molecule, resulting in limited surface hydrophobicity and poor emulsification properties, and this constraint curtails the application of egg white proteins in the emulsification systems [5]. Meanwhile, the constrained interfacial stability of egg white proteins at the oil–water interface poses a critical challenge in the development of egg white protein-based emulsion products. The optimization of egg white protein conformation via chemical or physical methods to augment protein interfacial adsorption and stability in emulsions is a crucial research topic currently under investigation [6,7].

Proanthocyanins, also recognized as condensed tannins, constitute a category of polymeric polyphenolic compounds formed by the linkage of flavonoid units and have a widespread presence in fruits, vegetables and flowers [8,9]. Proanthocyanins manifest a multitude of biological activities, including but not limited to anticancer, antidiabetic, and cardioprotective properties [9,10]. The abundant aromatic rings and phenolic hydroxyl groups, as hydrophilic and hydrophobic moieties within proanthocyanins, are perceived at a potential advantage for designing proanthocyanin-based emulsion biomaterials [8,11]. However, proanthocyanins are vulnerable to elevated temperature, exposure to oxygen, and light. Moreover, proanthocyanins undergo deprotonation in the acidic environment of the stomach, causing a decrease in the system’s electrostatic repulsion and stability [12]. Notably, the distinctive structural features of proanthocyanins bestow upon them unique interfacial activity, empowering them to regulate protein interactions at the oil–water interface. The polyphenol–protein conjugates are strategically situated with heightened density at the oil–water interface in comparison with unconjugated polyphenols, which not only amplifies the antioxidant potency but also fortifies the physical barrier effect [13]. Proanthocyanins have been validated to have a high affinity for binding with diverse proteins (e.g., salivary proteins, digestive enzymes, lysozyme). Hence, the formation of protein–proanthocyanin complexes can bring about a restructuring of the protein conformation, ultimately stabilizing the binary complexes of proteins and proanthocyanins and serving as a cornerstone for their stability within emulsions.

The conjugation of polyphenols with proteins can be delineated into irreversible covalent bonds and reversible non-covalent bonds [14]. Non-covalent interactions in proteins involve hydrogen bonding between hydrogen atom acceptors and polyphenolic hydroxyl groups, alongside the specific amino acid residues interacting with phenolic hydroxyls or aromatic rings via the ionic bonds, hydrophobic interactions and van der Waals forces [15]. Covalent interactions primarily revolve around the oxidative conversion of polyphenols into quinone compounds, which irreversibly interact with the thiols and amines of the proteins, forming C-S and C-N bonds [16]. In the current research landscape, the emphasis lies in the exploration of the individual impact of covalent or non-covalent binding modes on protein structure or functional properties. However, the interaction and structural ramifications of these two modification approaches on the conjugated compounds remain enigmatic, necessitating further investigation and elucidation of the underlying mechanisms responsible for these disparities. Moreover, the utilization of protein–polyphenol complexes in emulsions primarily revolves around emulsion stability, while investigations concerning the digestibility properties of emulsions are somewhat limited. Therefore, a promising area for further exploration lies in comparing the effects of protein–polyphenol covalent and non-covalent binding modes on the digestibility properties of emulsions.

In this research, we prepared covalent and non-covalent complexes of egg white proteins with proanthocyanins. The paper was developed in the following four aspects: (1) We explored the modulating effects of covalent and non-covalent interactions between proteins and proanthocyanins on protein conformation. (2) Furthermore, we studied the microstructural changes and interfacial properties of noncovalent and covalent complexes by wettability tests and atomic force microscopy (AFM). (3) By establishing scientific models for digestion kinetics and release kinetics, we predicted and assessed the dynamic digestion processes of proteins and oil in the emulsion. (4) Additionally, we performed correlation analyses to investigate the association between the physicochemical properties of proteins and the digestive properties of the emulsions. This study documents the application of binary complexes of egg white proteins and proanthocyanins in emulsions, providing new insights into the digestibility properties of emulsions and offering new technical approaches for analyzing dynamic changes during emulsion digestion processes.

## 2. Results and Discussion

### 2.1. Alterations in Protein Structure

**Figure 1:**
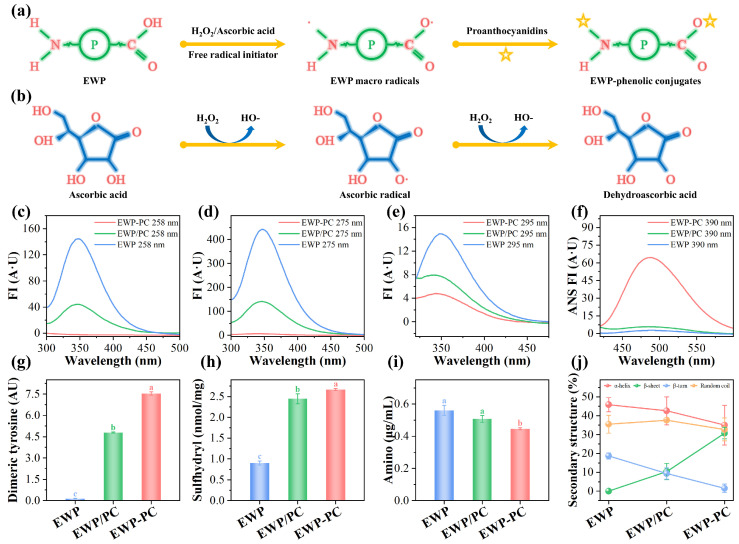
Schematic representation of proanthocyanins’ grafting mechanism (**a**,**b**). Intrinsic fluorescence (FI) and surface hydrophobicity: 258 nm FI (**c**); 275 nm FI (**d**); 295 nm FI (**e**); and ANS FI (**f**). Alterations to protein side-chain groups: Dimeric tyrosine (**g**); Free sulfhydryl group (**h**); Free amino group (**i**). Variation in protein secondary structure content (**j**). Note: The EWP, EWP/PC, and EWP-PC correspond to egg white protein, non-covalent complex, and covalent complex, respectively. ANS (8-anilino-1-naphthalene sulfonic acid FI (fluorescence intensity)) was utilized to signify the hydrophobicity of the protein surface. small letters showed significant differences between treatments (*p* < 0.05).

Intrinsic fluorescence spectroscopy is a prevalent method for investigating the protein–polyphenol interactions and alterations in the proteins’ tertiary structure [15]. Aromatic amino acids, including phenylalanine (258 nm), tyrosine (275 nm), and tryptophan (295 nm), assume a pivotal function as markers of intrinsic protein fluorescence. As depicted in Figure 1c–e, the introduction of proanthocyanins decreases the intrinsic fluorescence compared to the egg white proteins. This phenomenon might illustrate that the introduced proanthocyanins’ interaction with the egg white proteins induced the unfolding of the proteins’ tertiary structure [17,18]. The revelation of aromatic amino acids within the protein framework could decrease the intrinsic fluorescence of proteins in the solution microenvironments. In covalent complexes, there was a more noticeable decline in intrinsic fluorescence, driven by the augmented covalent interaction that promoted the unveiling of the hydrophobic protein domain [18,19].

The positioning of the hydrophobic amino acids within the internal and external regions of the protein is intricately linked to surface hydrophobicity, primarily regulated by hydrophobic interactions within the protein’s interior. As illustrated in Figure 1f, the incorporation of proanthocyanins resulted in an augmentation of the protein’s surface hydrophobicity in contrast to egg white protein alone. The interactions between the proteins and proanthocyanins might trigger the unfolding of the protein’s structure, which could prompt the migration of hydrophobic amino acids, such as phenylalanine, tyrosine, and tryptophan, from the protein’s interior to its surface, as supported by the findings of intrinsic fluorescence (Figure 1a–c) [19,20]. The increment in the quantity of hydrophobic amino acids situated on the protein’s surface contributed to an elevation of the protein’s surface hydrophobicity. In contrast to non-covalent complexes, covalent complexes manifested an enhancement in surface hydrophobicity. The prevalence of a robust covalent interaction mode enabled a greater exposition of hydrophobic amino acids on the protein’s surface, amplifying the surface hydrophobicity. Moreover, this outcome could be traced back to the covalent binding between egg white proteins and proanthocyanins in the redox system, which obstructed the anchoring of the hydrophilic groups, such as amine and mercaptan groups, to the protein’s surface [21].

In order to further investigate the impact of covalent and non-covalent interactions between egg white proteins and proanthocyanins on protein structure, the variations in protein secondary structure content were further explored. Obviously, the introduction of proanthocyanins could cause a decline in the content of α-helix and β-turn structure while concurrently leading to a rise in the quantity of β-sheet structure (Figure 1j). These variations suggested that interactions between egg white proteins and proanthocyanins could bring about structural changes in the protein, involving both deconstruction and unfolding [22]. Compared with non-covalent complexes, the covalent complexes demonstrated a marked reduction in the content of random coil, which illustrated the development of more organized secondary structures following the covalent binding. The content of α-helix and β-turn structures substantially decreased, while the content of β-sheet structure dramatically increased in the covalent complexes. The presence of both disulfide and hydrogen bonds might be attributed to the change of α-helix structure content in the covalent complexes, while the presence of hydrogen bonding interactions tended to promote the formation of β-sheet structures in the covalent complexes [18]. The elevated β-sheet content was instrumental in alignment orderliness, which was of utmost importance for fostering interactions between the protein–lipid and protein–protein interactions [23]. The outcomes highlighted the considerable potential of the covalent complexes in advancing emulsion performance.

### 2.2. Variations of Amino Acid Side Chain Groups

Dimeric tyrosine emerges from the covalent linkage of adjacent tyrosine phenoxy radicals. These radicals arise from the oxidation of hydroxyl groups within the aromatic ring of the tyrosine. The incorporation of the proanthocyanins markedly augmented the synthesis of the dimeric tyrosine (Figure 1g). Within the redox milieu, the culmination of dimeric tyrosine formation was attributed to the existence of hydrogen peroxide [24]. Hydrogen peroxide catalytically fostered the covalent crosslinking, occurring intra- or intermolecularly amongst the proteins, via the direct oxidation of aromatic rings within two tyrosine molecules [25]. This procedure culminated in the deprotonation of the phenolic hydroxyl groups, potentially giving rise to the considerable establishment of a covalent complex encompassing phenoxy radicals [26].

Free sulfhydryl groups manifest as remarkably chemically active functional units, actively participating in diverse chemical reactions within the food systems [27,28]. Their direct contribution influences the functional properties, redox state, and eventual composition of proteins found in the food products [28]. The data illustrated in Figure 1h underscored that the utilization of proanthocyanins enhanced the extent of the free sulfhydryl groups. In light of the intrinsic fluorescence and surface hydrophobicity analyses, it was plausible to infer that proanthocyanins facilitated protein unwinding, potentially inducing the intramolecular disulfide linkages’ perturbation [27]. This could provide an explanation for why the addition of proanthocyanins could increase the content of the free sulfhydryl groups. In comparison with the non-covalent complexes, covalent complexes exhibited a higher presence of free sulfhydryl groups. The principal impetus behind this phenomenon was instigated by the redox system and the effect of ultrasound cavitation. As a consequence of these factors, the protein conformations unwound, exposing the free sulfhydryl groups in the interior of the egg white proteins [27,29].

The ophthalmic aldehyde (OPA) method is chiefly utilized to discern the N-terminal residues and α-amino or ε-amin of the lysine in the proteins, thereby quantifying the content of amino groups. The quantifying free amino group content provides insight into the binding interaction between polyphenolics and proteins. Figure 1a,b illustrate the mechanism of covalent grafting of proteins by proanthocyanins within the free radical system. Figure 1i visually captures the fluctuations in free amino acid contents prior to and following the addition of proanthocyanins. Egg white proteins and proanthocyanins culminated in the formation of non-covalent complexes, which reduced the content of free amino groups. The covalent complexes exhibited a greater capability to markedly diminish free amino groups. This phenomenon could be primarily attributed to the covalent modification of proanthocyanins, characterized by the interaction between oxidized phenolic groups and nucleophilic amino acid residues, particularly focusing on cysteine and lysine residues located within protein side chains [30]. Moreover, another conceivable factor was the sono-chemical effect of ultrasound, wherein ultrasound instigated the generation of additional hydroxyl radicals [31]. It is conceivable that a larger number of unbound amino groups could potentially undergo oxidation by the hydroxyl radicals, leading to the formation of protein structures with heightened activity [31]. This, in turn, could trigger more reactions between free amino groups and proanthocyanins, resulting in the formation of C-N bonds.

### 2.3. Fourier Transform Infrared Spectroscopy (FTIR) and X-ray Diffraction (XRD) Analysis

**Figure 2:**
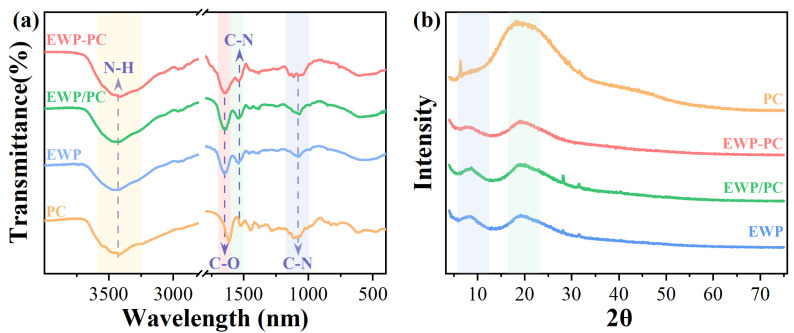
FTIR (**a**) and XRD (**b**) spectrum. Note: The PC, EWP, EWP/PC, and EWP–PC correspond to proanthocyanins, egg white protein, non-covalent complex, and covalent complex, respectively.

The application of FTIR allowed for the detection of interactions between the egg white protein–proanthocyanins complexes via covalent and non-covalent associations. As depicted in Figure 2a, the absorption peak of N-H bonds at 3425 cm^−1^ displayed as sharper and narrower after the introduction of proanthocyanins. This phenomenon indicated the presence of hydrogen bonding interactions within covalent and non-covalent complexes [15]. At the absorption wavelength of 1652 cm^−1^, aligning with the absorption peak of the C-O bond, a distinct alteration was discernible induced by the proanthocyanins [18]. The peak position experienced a redshift transitioning from 1652 to 1650 and 1648 cm^−1^, accompanied by a gradual reduction in peak intensity within both non-covalent and covalent complexes. The absorption peak situated at 1540 cm^−1^ was indicative of the C-N bond absorption [32]. In the covalent complexes, this absorption peak underwent significant attenuation, indicating the capacity of proanthocyanins to foster covalent interactions with more free amino groups in the proteins [18]. The absorption peak at 1080 cm^−1^ further corroborated the above findings, aligning with the analysis obtained from the free amino group.

The utilization of XRD is a customary method to analyze variations in the crystal structures of polyphenols, proteins, and other substantial molecular biopolymers [33]. As shown in Figure 2b, the characteristic peaks of the pure proanthocyanins were observed at 6.3° and 19.1°. The characteristic peak positions of egg white proteins were chiefly at around 8.2° and 18.9°. The XRD pattern of proanthocyanins combined with the proteins exhibited no distinct sharp peaks, signifying the formation of a novel complex wherein the proanthocyanins transitioned from a crystalline structure to an amorphous structure [34]. It was noteworthy that the intensity of the characteristic peak of the covalent complex at 8.2° experienced a notable reduction. This indicated that the protein became smaller and gained greater uniformity following the formation of the covalent complex [33].

### 2.4. Wettability and AFM Analysis

**Figure 3:**
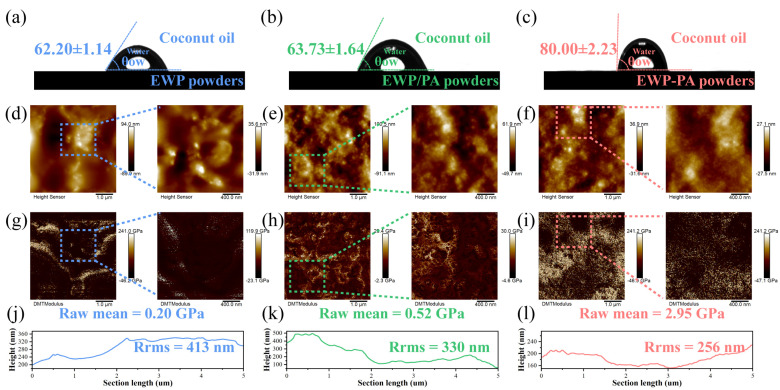
Contact angle (**a**–**c**), AFM images (**d**–**f**), rigidity (**g**–**i**), and height distribution (**j**–**l**). RMS is root-mean-square height. The Raw mean represents a rigid within the region. Note: The EWP, EWP/PC, and EWP–PC correspond to egg white protein, non-covalent complex, and covalent complex, respectively. The enlarged images of the dotted squares in (**d**–**f**) are presented on the right.

The wettability of proteins between the oil and aqueous phases can be articulated by the three-phase contact angle (θ), which is closely linked to interfacial properties for assessing the behavior of hydrophobicity for the surface film [35]. During the process of emulsification, the proteins are concurrently wetted by the continuous and dispersed phases, which are influenced by the cohesive and adhesive forces between the water phase and the surface film [35,36]. As depicted in Figure 3a,b, the θ values of the proteins and non-covalent complexes were consistently below 90°, implying their propensity to stabilize the oil-in-water emulsions. Insights from prior studies have illuminated that a contact angle close to 90° is valuable for enhancing the stability of Pickering emulsion. Notably, the θ value of EWP–PA powders measured 80.00° ± 2.23°, indicating that the covalent complexes significantly optimized the wettability of the proteins in accordance with surface hydrophobicity (Figure 3c). Following the coating of proanthocyanins onto the proteins, the phenolic hydroxyl groups of the proanthocyanins interacted with the proteins’ hydroxyl, carboxyl, and amino groups. Thus, the hydrophilic groups on the protein’s surface were diminished, which further enhanced the surface hydrophobicity. This phenomenon illustrated that covalent complexes could exhibit effective adsorption and accumulation on the droplet’s surface, functioning as the steric barrier that hindered the condensation of oil droplets within emulsions.

AFM functions adeptly in elucidating surface microstructure and Young’s modulus. As illustrated in Figure 3d–f, the surface morphology of the proteins is demonstrated. Egg white protein itself exhibited a globular microstructure. Upon the introduction of proanthocyanins, the particle size distribution of the protein became more uniform and smaller. The rigidity of the proteins was assessed using Young’s modulus obtained through AFM [37]. The existence of the proanthocyanins yielded an elevated level of rigidity in the proteins, particularly prominent when covalent linkages were formed between proanthocyanins and proteins (Figure 3g–i). Prior research findings suggested that the proteins with higher rigidity were less prone to deformation at the oil–water interface, thus averting the rupture of the interfacial film [23]. This improved stability facilitated more robust and secure protein adsorption at the oil–water interface. Covalent or non-covalent complexes could significantly reduce the roughness of the proteins (Figure 3j–l). The roughness of the covalent complexes exhibited a more pronounced reduction. We ascribed the roughness alteration mainly to the wettability modification, which also correlated with the changes in the size and dispersion of the constituent proteins.

### 2.5. Microstructure Observation

**Figure 4:**
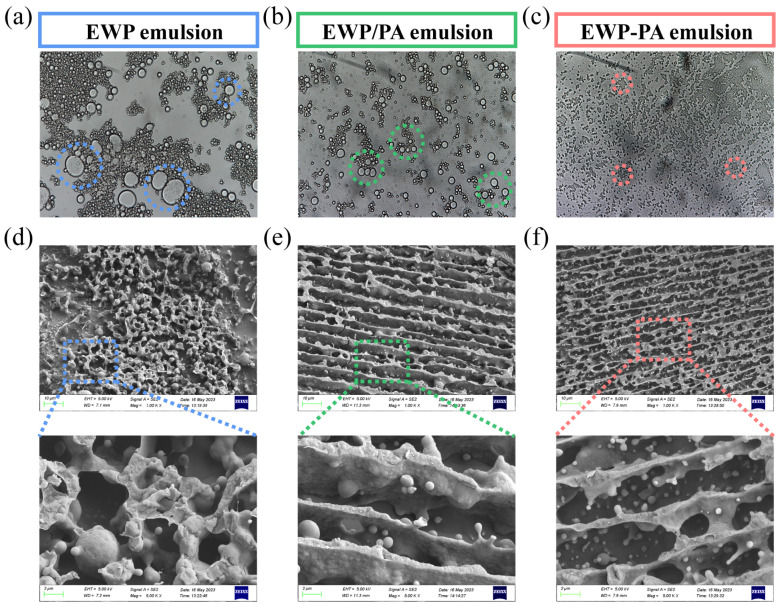
Optical microscopy images (**a**–**c**) and Cryo-SEM photographs (**d**–**f**). Note: The dotted colored circles in the image highlight the aggregation of large droplets.

The emulsion’s appearance and droplet distribution could be visually revealed by the optical morphology (Figure 4a–c). The emulsion droplet uniformly presented spherical shapes, pointing towards the protein adhesion to the oil–water interface, contributing to the preservation of droplet stability. Typical flocculation clustering of oil droplets was observed in egg white proteins’ stabilized emulsion. However, proanthocyanins induced a considerable decrease in droplet aggregation. This phenomenon was primarily attributed to the reinforced complex structure that fortified the oil–water interface of the emulsion against the wetting effect [38]. The covalent complex stabilized emulsion manifested a precise and uniform size distribution of the emulsion droplets, potentially influenced by the appropriate hydrophobic–hydrophilic balance of the proteins [39]. Meanwhile, the higher hydrophobic interaction functioned as a safeguard against the aggregation of the emulsion droplets. The covalent interaction between proanthocyanins and proteins potentially bolstered the protein emulsification attributes, promoting uniform droplet distribution [40,41].

The representative cryo-scanning electron microscopy (cryo-SEM) images, along with magnified images, are employed to provide deeper insights into the microstructure of the emulsion in Figure 4d–f. The observations hinted that, within the emulsions’ stabilized egg white proteins, the proteins portrayed a disorganized and irregular distribution. Generally, the construction of the network within the continuous phase provided a favorable component for augmenting emulsion stability. In the emulsions stabilized by the complexes, the protein network was uniformly distributed and tightly interconnected. Upon the formation of the non-covalent and covalent complexes, the optimized steric hindrance could facilitate the development of a systematically organized network, leading to structural stability [42]. In the covalent complex stabilized emulsion, the network architecture exhibited greater density and compactness, amplifying its affinity for the oil droplets and inter-droplet friction, which proficiently impeded droplet coalescence and upheld the emulsion’s stability.

### 2.6. Emulsion Stability

**Figure 5:**
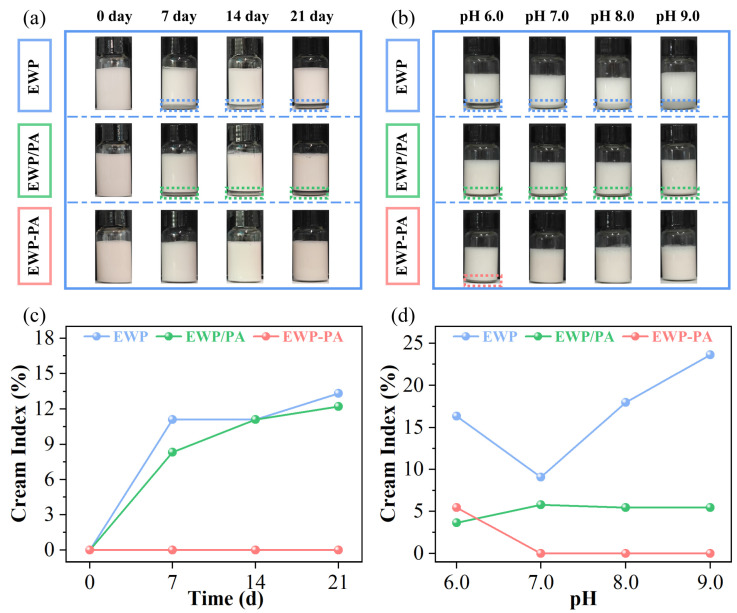
The overall appearance of emulsion storage stability (**a**) and pH stability (**b**). The creaming index for storage stability (**c**), pH stability (**d**). Note: The dotted colored squares in the image highlight the creaming phenomenon of the emulsion.

The storage stability of the emulsion holds profound significance in the context of its applications. The instability of emulsions following storage at room temperature was monitored and photographed (Figure 5a). Each freshly prepared emulsion showcased a milky white appearance with a fine and uniform texture. Over time, the EWP emulsion displayed a progressive emergence of the phase separation. The upsurge in creaming index (CI) further confirmed that the emulsification of the EWP-stabilized emulsion intensified with the extension of time (Figure 5c). This primarily originated from the comparatively feeble interfacial attributes of egg white proteins, rendering them less proficient at upholding extended stability after their adsorption at the oil–water interface [5]. After storage of 30 days, the covalent complex stabilized emulsion exhibited good storage stability. Upon the covalent binding of proteins with proanthocyanins, the optimization of the three-phase contact angle enabled the protein’s stable adsorption at the oil–water interface. The rigid structure resulting from the covalent binding of proteins with proanthocyanins was less susceptible to the effects of wetting phenomena [38].

In various food processing and matrix conditions, it is imperative for emulsions to maintain stability across different pH levels. We conducted a study to investigate the impact of pH on the stability of emulsions. As illustrated in Figure 5b, emulsions stabilized solely by the proteins failed to retain stability across diverse pH conditions, exhibiting differing degrees of creaming or phase separation. Meanwhile, the introduction of proanthocyanins substantially amplified the stability of the emulsion across diverse pH ranges, effectively suppressing creaming occurrences (Figure 5d). The covalent complex benefited from the robust steric hindrance and electrostatic repulsion, which could proficiently prevent the occurrence of flocculation [43].

### 2.7. Emulsion Digestive Kinetics and FFA Release Kinetics

**Figure 6:**
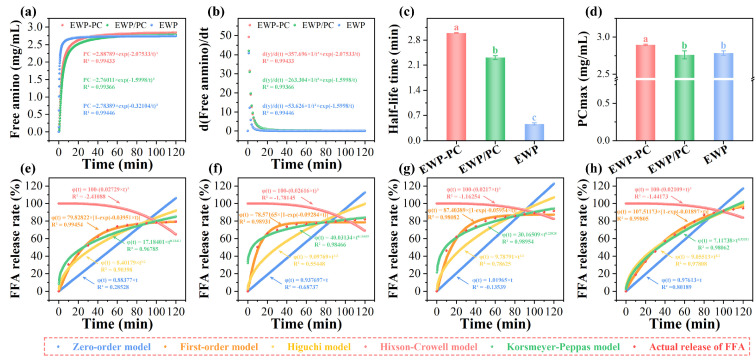
Fitted curves for emulsion digestion kinetics (**a**) corresponding curve derivation (**b**), half−life (**c**), and maximum peptide concentration (**d**). FFA release kinetics of egg white proteins’ stabilized emulsion (**e**), non−covalent stabilized emulsion (**f**), covalent complex stabilized emulsion (**g**), and oil (**h**). small letters showed significant differences between treatments (*p* < 0.05).

The mechanism of protein digestion within the emulsions, as well as the liberation of amino acids, was subjected to kinetics investigation. The outcomes of gastric digestion kinetics are delineated in Figure 6a–d. PCmax, maximum digestion rate, and half-life time are crucial indicators for assessing the level and rate of digestion. The gastric protease digestion curve obtained through fitting the peptide concentration and the calculated digestion rate curve are depicted. After the addition of pepsin, the rate of protein digestion increased rapidly initially but then decreased rapidly. This might be due to the decrease in the accessibility of pepsin, resulting in a sharp decrease in the rate of digestion. In the covalent complex, the release of protein peptides could proceed gradually, eventually reaching the maximum extent of release. The covalent binding of proteins to polyphenols had the potential to increase the degree of digestion of proteins in the stomach, which might facilitate the release of free amino acids for digestion and absorption by the body [44].

Figure 6e–h illustrate the release kinetics of FFA in the emulsions, highlighting the suitability of both first-order and Korsmeyer–Peppas models for explaining the dynamic release process. The first-order kinetics model was particularly well-suited for elucidating the release of FFA from the oil and emulsions stabilized by egg white proteins and the non-covalent complex (Table 1). This observation indicated that the release of FFA gradually diminished with time, eventually reaching an equilibrium state. The “n” value obtained by the Korsmeyer–Peppas model of the oil was 0.55551, signifying that the release behavior corresponded to abnormal transport involving a combination of Fickian diffusion and case-II transport [45]. In all emulsions, the “n” value is below 0.45, implying the Fickian diffusion kinetics. Furthermore, the release curve aligned well with the Higuchi plane diffusion equation, expressed as Q = 9.05513 × t^1/2^, with an R^2^ value exceeding 0.97. This suggested the possible existence of the Higuchi plane diffusion equation as a release mechanism for the release of FFA from the oil.

### 2.8. Correlation Analysis

**Figure 7:**
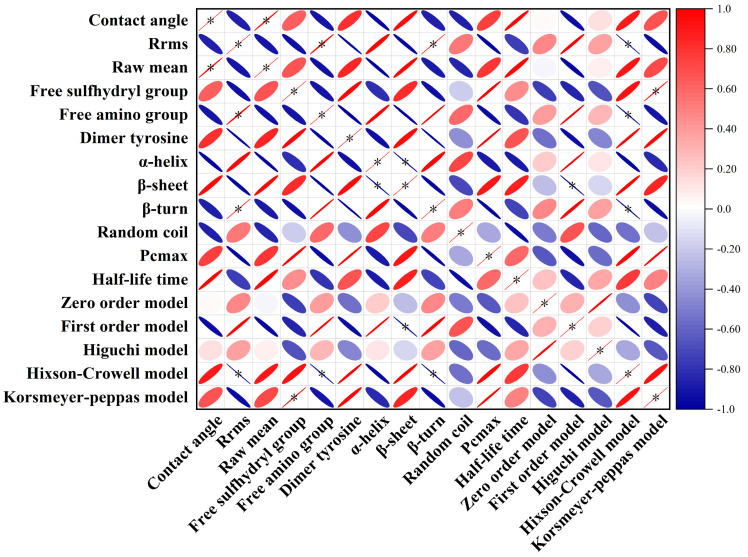
Correlation analysis.

Correlation analysis was employed to investigate the relationship between the physicochemical attributes of proteins, emulsion stability, and the digestive and release kinetics (Figure 7). There was a conspicuous correlation in terms of contact angle and average protein size. These disparities primarily stemmed from the covalent or non-covalent binding to optimize the hydrophilic–hydrophobic balance of the proteins, which favorably yielded a more consistent protein size [33,39]. Additionally, we observed significant variations, in terms of Hixcon–Crowell, with free amino groups and structural rigidity. Furthermore, distinctions emerged regarding the Korsmeyer–Peppas model and the presence of free sulfhydryl groups. This suggested that the approach of covalent or non–covalent binding could influence the content of free sulfhydryl groups, subsequently affecting the model fitting for release kinetics.

## 3. Materials and Methods

### 3.1. Materials

Fresh eggs and coconut oil were acquired from the local supermarket (Changchun, China). Proanthocyanins (≥95%), Nile red, and Nile blue were obtained from Yuanye Bio-Technology Co., Ltd. (Shanghai, China). Ascorbic acid (≥98%) was sourced from Aladdin Bio-Chem Technology Co., Ltd. (Shanghai, China). Pepsin from porcine gastric mucosa (P7000, ≥250 U/mg), bile salts, lipase from porcine pancreas (L3126, 100−500 U/mg), and pancreatin from porcine pancreas (P1625, ≥3 × USP) were acquired from Sigma Aldrich (Hamburg, Germany). All the other reagents and solvents were standardized at analytical levels.

### 3.2. Formulation of Egg White Protein–Proanthocyanin Complexes

Egg white proteins were isolated from fresh eggs and then subjected to intensive stirring for 30 min at pH 5.0 according to a slightly modified version of the previously established method [3]. The isolated egg white mixture, once separated, underwent centrifugation (H2050R, Cence Co., Ltd., Changsha, China) at 10,000× *g* for a duration of 15 min to ensure the separation of unwanted and insoluble components. The resulting supernatant was diluted with deionized water until a protein concentration of 10 mg/mL (BCA kit, Beyotime, Shanghai, China). The egg white solution corresponding to the protein concentration was readjusted to pH 7.0 as a control.

The formation of the egg white protein–proanthocyanin covalent complex was accomplished through an ultrasonic-assisted free radical methodology. Initially, H_2_O_2_ (2.0 mL, 10 mM) and 0.5 g of ascorbic acid were introduced to the egg white protein solution of 200 mL (pH 7.0, 10 mg/mL). The resultant mixture solution was subjected to an incubation of 2 h in a 25 °C thermostatic water bath shaker (100 r/min). Subsequently, proanthocyanins were dissolved in the redox system constructed above and subjected to SCIENTZ-IID ultrasonic reactor (Ningbo Xinzhi organism Co., Ltd., Ningbo, China) incubation for 1 h (40 kHz, 300 W, 5/3 s on/off). As for the non-covalent complex, proanthocyanins (0.2 g) were incorporated into the egg white protein solution of 200 mL (pH 7.0, 10 mg/mL). The obtained mixture solution was stirred vigorously at 500 rpm for 1 h, culminating in the creation of the non-covalent complex.

### 3.3. Determination of Protein Structural Modifications

#### 3.3.1. Intrinsic Fluorescence Spectroscopy

Each protein sample (0.5 mg/mL) was prepared with deionized water at the corresponding pH. Emission spectra within the wavelength range 300 to 450 nm were systematically documented at the excitation wavelengths: 258 nm, 275 nm, and 295 nm [46]. Equally, the slit widths for both excitation and emission were uniformly established at 5 nm.

#### 3.3.2. Surface Hydrophobicity

The evaluation of surface hydrophobicity for each protein sample was conducted utilizing the fluorescent probe 8-anilino-1-naphthalene sulfonic acid (ANS). Briefly, the ANS reagent (20 μL, 8 mM) was promptly added to the protein sample solution (4 mL, 0.5 mg/mL), followed by incubation in darkness (25 °C, 15 min). The excitation of the emission spectrum transpired at 390 nm, and the resultant emission wavelengths spanning 400 to 600 nm were documented in triplicate [47].

#### 3.3.3. Circular Dichroism (CD) Spectroscopy

The CD spectra of a 200 μL protein solution (0.5 mg/mL) were captured at 25 °C. The spectral range covered 190 to 250 nm, with a resolution of 1 nm. The scanning speed was consistently sustained at 300 nm/min. The identification of characteristic secondary structures was executed through the utilization of the online BeStSel software (Version 1.3.230210) [48].

### 3.4. Alterations of Amino Acid Side Chain Groups in Proteins

#### 3.4.1. Dimer Tyrosine

Each protein sample was diluted to 1 mg/mL with a 0.1 mM phosphate buffer solution (pH 7.4). Fluorescence measurements (F-7100, Hitachi, Tokyo, Japan) were conducted using an excitation wavelength of 324 nm and an emission wavelength of 420 nm. The obtained fluorescence intensity, when normalized by the corresponding protein concentration, represented the dimer tyrosine content of the sample.

#### 3.4.2. Free Sulfhydryl Group

Each protein sample was blended with Tris-glycine buffer (0.086 M Tris, 0.09 M glycine, and 4 mM EDTA, pH 8.0) at a volume ratio of 1:2. The mixtures were combined with 50 μL of Ellman’s reagent (4 mg/mL of DTNB in the 1X phosphate buffer) and then incubated at 25 °C for 30 min in darkness. The absorbance of the protein samples was quantified at 412 nm via the UV-vis spectrophotometer (UV-2550, Shimadzu, Kyoto, Japan) [47]. The buffer without protein samples was employed as the blank control. The free sulfhydryl groups were conducted based on the following formula:

Free sulfhydryl group (nmol/mg) = A_412_ × 73.53 × D/C, (1)

where A_412_ represents the sample’s absorbance at 412 nm, D denotes the dilution factor, and C corresponds to the protein concentration of the sample supernatant (mg/mL).

#### 3.4.3. Free Amino Group

The quantification of free amino groups was conducted following the OPA method with minor adjustments [49]. For the OPA reagent preparation, 80 mg of OPA was dissolved in 2 mL of methanol, and then 5.0 mL of 20% (*w*/*v*) sodium dodecyl sulfate, 200 μL of β-mercapto-ethanol, 50 mL of 0.1 M sodium borate buffer, and deionized water were sequentially added to complete a final volume of 100 mL. The protein sample (1 mg/mL) was combined with OPA reagent at a volume ratio of 1:2 and incubated for 2 min (25 °C). The absorbance was measured at 340 nm via the UV-vis spectrophotometer. The quantification of free amino group contents was executed by applying the standard calibration curve of L-leucine (0.01–0.07 mg/mL) (R^2^ ≥ 0.999).

### 3.5. FTIR

The lyophilized powders from each sample were pressed in conjunction with KBr into thin tablets at the mass ratio of 1:100. Spectra were acquired within the wavelength range of 4000 to 400 cm^−1^ with a spectral resolution of 4 cm^−1^ [2].

### 3.6. XRD

The X-ray diffractometer (Bruker D8, ADVANCE, Billerica, Germany) was utilized to execute a scanning operation on the protein lyophilized powders, employing an accelerating voltage of 40 kV and a current of 40 mA with a Cu anode. The scanning of 2θ angles spanned from 4 to 75°, and this process was carried out with precision at a scanning speed of 0.05°/min.

### 3.7. Three-Phase Contact Angle

The evaluation of three-phase contact angles was undertaken via the Dataphysics OCA20 contact angle goniometer [50]. Through a compression process, the protein lyophilized powders (0.15 g) were converted into tablets and then situated on optical glass vessels containing coconut oil. The Young–Laplace equation was employed for fitting contact angle values. A minimum of two droplets were measured for each tablet and evaluated per sample at least three times to ascertain the average contact angle.

### 3.8. AFM

The protein samples’ morphology and rigidity (Young’s modulus) were examined utilizing atomic force microscopy (AFM, Bruker, Dimension Icon, Berlin, Germany) [37]. The protein samples were deposited onto the cleaved mica sheet and then dried at ambient temperature. Experiments were executed using the PeakForce Quantitative Nanomechanical (PeakForce QNM) mode. Analysis of images and average rigidity value were conducted using Nanoscope Software (Version 3.00).

### 3.9. Preparation of Emulsion

Protein sample solutions (10 mL, 10 mg/mL protein) and coconut oil (30 mL) were subjected to homogenization (12,000 rpm, 2 min) to generate the crude emulsion, which was then promptly relocated to the ultrasonic reactor for an incubation duration of 5 min (40 kHz, 380 W, 5/3 s on/off) to obtain the fine emulsion. Furthermore, the freshly prepared emulsions were maintained at ambient temperature (25 °C) until further examination.

### 3.10. Microscopic Observations

#### 3.10.1. Optical Microscope

The fresh emulsions were carefully placed onto glass slides, enabling the examination of droplet size distribution using an optical microscope (Ti2, Nikon, Tokyo, Japan).

#### 3.10.2. Cryo-SEM

The emulsion samples were positioned onto the copper support and submerged in liquid nitrogen for plunge-freezing (−160 °C). Once the freeze-drying process for water sublimation (−90 °C, 15 min) was concluded, samples were fractured with the scalpel blade. The sectioned surface of emulsion samples was coated with the sputtered gold and then introduced into the observation chamber within a cold platform module (−125 °C) (Cryo-SEM, ZEISS, Munich, Germany).

### 3.11. Stability Evaluation

#### 3.11.1. Storage Stability

The prepared emulsions were transferred into 5 mL glass vials and then stored for a period of 28 days at ambient temperature (25 °C). The calculation of the creaming index (CI) involved assessing the clear liquid’s height (H_0_) and the overall height of the emulsion (H_1_), with the calculation formula being articulated as follows:

CI = H_0_/H_1_, (2)

#### 3.11.2. pH Stability

The freshly prepared emulsion samples were modulated to pH 6.0, 7.0, 8.0, and 9.0 by 1 M NaOH or HCl. Following this adjustment, the emulsion samples were permitted to reach a state of stability at ambient temperature, preparing it for the stability analysis and creaming index analysis via Equation (2).

### 3.12. Emulsion Digestion Kinetics

The emulsion samples (2.0 mL) were digested with simulated gastric fluid (SGF, 18 mL, containing 4.8 mg/mL pepsin), and the resulting mixtures were adeptly adjusted to pH 2.0. The process of the simulated gastric digestion was upheld within thermostatic conditions (37 °C, 120 rpm). Simulated gastric digestive samples (40 μL) were extracted at multiple time points (0, 5, 10, 15, 20, 25, 30, 40, 50, 60, 70, 80, 90, 100, 110, and 120 min), and then OPA reagent (160 μL) was incorporated to investigate the free amino group content. The release profile of free amino groups was modeled using a first-order kinetic equation. “PC” represents the released free amino acid content. “PCmax” signifies the ultimate level of proteolysis during indefinite digestion. The half-life time is the period necessary to yield the number of peptides equal to half PCmax.

PC = PCmax × exp(−B/time),(3)

where B = (half-life time) × ln2,(4)

d(PC)/d(time) = 60 × PCmax × B × 1/(time)2 × exp(−B/time),(5)

### 3.13. FFA Release Kinetics

After repeating gastric digestion without sample extraction, the gastric digesta was regulated to pH 7.0 and mixed with 20 mL of simulated intestinal fluid (SIF, comprising 10 mg/mL bile salts, 4.8 mg/mL pancreatin, and 4.8 mg/mL lipase, pH 7.0) readjusted to pH 7.0. Then, the obtained mixtures were subjected to a 4-h incubation in a shaker (37 °C, 120 rpm). During the simulated intestinal digestion process, the pH level was sustained at 7.0 by adding 1.0 M NaOH to effectively neutralize the released FFA. The calculation of the released FFA quantity could be accomplished as outlined below.

Five distinctive release kinetic models were employed to elucidate the release mechanisms linked to FFA in the emulsion: the zero-order model, first-order model, Higuchi model, Hixson−Crowell model, and Korsmeyer–Peppas model [45,51]. The optimal model for portraying the release kinetics of FFA was identified by choosing the model with the highest coefficient of determination (R^2^) [51]. Correlation analysis was leveraged to scrutinize the associations between digestion kinetics, release kinetics, and modifications in the attributes of covalent or non-covalent complexes. Data processing and correlation analysis were conducted using Origin software, and the outcomes were visually represented in the form of a heatmap.

Zero order model: φ(t) = k_0_ × t, (6)

First order model: φ(t) = k_1_[1−exp (−k_2_ × t)] (7)

Higuchi model: φ(t) = k_3_ × t^1/2^ (8)

Hixson–Crowell model: φ(t) = 100 − (k_4_ × t)^3^ (9)

Korsmeyer–Peppas model: φ(t) = k_5_ × t^n^ (10)

where “t” signifies the duration of intestinal digestion time (min), φ(t) represents the total cumulative FFA level at the time “t”, k_0_, k_1_, k_2_, k_3_, k_4_, and k_5_ are rate constants, while “n” represents the release exponent.

### 3.14. Statistical Analysis

All experiments were conducted in triplicate, and the results are presented as the mean ± standard deviation. Statistical analysis was performed using a one-way analysis of variance (ANOVA), with statistical significance set at a threshold of *p* < 0.05.

## 4. Conclusions

To summarize, this study elucidated the impacts of the covalent and non-covalent modifications of proanthocyanins on protein properties. Proanthocyanins tuned the protein’s secondary and tertiary structure. The covalent interactions, with their greater intensity, markedly enhanced the surface hydrophobic, interface wettability, and structural rigidity of the proteins, yielding excellent properties that supported emulsion stability. Inspection of digestion kinetics revealed that emulsions stabilized by the covalent complex not only achieved optimal protein digestion but also facilitated gradual peptide release. FFA release kinetics demonstrated that both first-order and Korsmeyer–Peppas models effectively described the release patterns. Correlation analysis illustrated an association between the Korsmeyer–Peppas kinetic model and the content of free sulfhydryl groups. This research offers valuable insights into designing and formulating emulsions stabilized by the protein–polyphenol complexes, as well as innovative approaches for analyzing the dynamic process of emulsion digestion.

## Figures and Tables

**Table 1:** Fitting data of FFA release obtained using various mathematical models.

Sample	Zero Order (R^2^)	First Order (R^2^)	Higuchi (R^2^)	Hixson–Corwell (R^2^)	Korsmeyer–Peppas (R^2^)
EWP emulsion	0.28528	0.99454	0.90398	−2.41088	0.96785
EWP/PA emulsion	−0.68737	0.98931	0.55448	−1.78145	0.98466
EWP–PA emulsion	−0.13539	0.98082	0.78625	−1.16254	0.98954
Oil	0.80189	0.99805	0.97808	−1.44173	0.98062

## Data Availability

The raw data supporting the conclusions of this article will be made available by the authors on request.
